# Local Progression Kinetics of Geographic Atrophy Depends Upon the Border Location

**DOI:** 10.1167/iovs.62.13.28

**Published:** 2021-10-28

**Authors:** Liangbo L. Shen, Mengyuan Sun, Aneesha Ahluwalia, Michael M. Park, Benjamin K. Young, Lucian V. Del Priore

**Affiliations:** 1 Department of Ophthalmology, University of California, San Francisco, San Francisco, California, United States; 2 Institute of Cardiovascular Diseases, Gladstone Institute, San Francisco, California, United States; 3 Byers Eye Institute, Department of Ophthalmology, Stanford University School of Medicine, Palo Alto, California, United States; 4 Department of Ophthalmology, New York Eye and Ear Infirmary of Mount Sinai, New York, New York, United States; 5 Department of Ophthalmology and Visual Science, Kellogg Eye Center, University of Michigan Medical School, Ann Arbor, Michigan, United States; 6 Department of Ophthalmology and Visual Science, Yale School of Medicine, New Haven, Connecticut, United States

**Keywords:** age-related macular degeneration, geographic atrophy, natural history, color fundus photography

## Abstract

**Purpose:**

To assess the influence of lesion morphology and location on geographic atrophy (GA) growth rate.

**Methods:**

We manually delineated GA on color fundus photographs of 237 eyes in the Age-Related Eye Disease Study. We calculated local border expansion rate (BER) as the linear distance that a point on the GA border traveled over 1 year based on a Euclidean distance map. Eye-specific BER was defined as the mean local BER of all points on the GA border in an eye. The percentage area affected by GA was defined as the GA area divided by the total retinal area in the region.

**Results:**

GA enlarged 1.51 ± 1.96 mm^2^ in area and 0.13 ± 0.11 mm in distance over 1 year. The GA area growth rate (mm^2^/y) was associated with the baseline GA area (*P* < 0.001), perimeter (*P* < 0.001), lesion number (*P* < 0.001), and circularity index (*P* < 0.001); in contrast, eye-specific BER (mm/y) was not significantly associated with any of these factors. As the retinal eccentricity increased from 0 to 3.5 mm, the local BER increased from 0.10 to 0.24 mm/y (*P* < 0.001); in contrast, the percentage of area affected by GA decreased from 49.3% to 2.3%.

**Conclusions:**

Using distance-based measurements allows GA progression evaluation without significant confounding effects from baseline GA morphology. Local GA progression rates increased as a function of retinal eccentricity within the macula which is opposite of the trend for GA distribution, suggesting that GA initiation and enlargement may be mediated by different biological processes.

Geographic atrophy (GA) secondary to nonexudative age-related macular degeneration (AMD) is characterized by well-demarcated borders of atrophic areas in the macula, affecting over 5 million people worldwide.^1^ Eyes with GA have progressive degeneration of photoreceptors, retinal pigment epithelium (RPE), and choriocapillaris in the setting of extracellular deposits.^2^^,^^3^ GA lesions have various shapes^4^ and typically spare the fovea until later stages.^5^^–^^8^ The underlying mechanisms of GA expansion, the occurrence of various lesion shapes, and the foveal sparing phenomenon remain unclear.

Currently, there are no approved therapies for reversing or slowing GA progression, but several early-phase clinical trials have reported promising results, with many other trials underway.^9^^–^^12^ The growth rate of total GA size is the primary endpoint in most previous clinical trials.^13^ However, the growth rates of GA area have varied widely across different studies, ranging from 0.53 to 2.79 mm^2^/y.^4^^,^^14^^–^^16^ GA area growth rate (in mm^2^/y) is associated with baseline morphological factors, including GA area, lesion number, and circularity index.^4^^,^^14^^,^^16^^–^^23^ Several studies have shown that the growth rate of GA effective radius (equivalent to square root of area, expressed in in mm/y) was independent of baseline size and the elapsed time but still dependent on lesion number and circularity.^14^^,^^17^^,^^18^^,^^27^ Recently, we demonstrated that, after adjustment for GA perimeter, GA growth rate was uncorrelated with baseline GA area, lesion number, and circularity.^23^ These findings imply that the linear expansion rate of the leading edge of GA is independent of lesion morphology. However, these previous studies did not directly measure the linear border expansion rate (BER) of GA, so subsequent studies are required to investigate this hypothesis further.

The GA growth rate has been associated with lesion location.^5^^,^^8^^,^^18^^–^^20^^,^^24^^–^^26^ The growth rate of total GA effective radius was approximately 30% to 60% higher in non-central GA than in central GA.^5^^,^^8^^,^^18^^–^^20^^,^^24^^–^^26^ Lindner et al.^5^ found a faster atrophy progression toward the periphery than toward the fovea. These findings imply that GA growth rate varies by topographic location, consistent with the foveal sparing phenomenon. Our previous meta-analysis of study-level data demonstrated that GA growth rate varied progressively as a function of retinal eccentricity and that the topographic variation of the GA growth rate could give rise to various GA shapes based on modeling.^27^ However, due to the lack of patient-level data in our previous meta-analysis, these findings must be validated by quantifying GA BERs across different topographic locations in individual patients.

The present study aimed to investigate the impact of topographic location on the local progression rate of GA using data from the Age-Related Eye Disease Study (AREDS). We employed a Euclidean distance map to measure the GA BER (in the linear distance) as proposed by Uji et al.^28^ We also compared the topographic variation in GA growth rate and GA distribution and investigated the hypothesis that the GA BER is uncorrelated with baseline GA morphological factors (i.e., GA area, lesion number, and circularity).

## Methods

### Study Participants and Image Grading Procedure

We obtained color fundus photographs (CFPs) and clinical data from the original AREDS via the database of Genotypes and Phenotypes (dbGaP Study Accession: phs000001.v3.p1)^29^ after obtaining approval for authorized access. The Yale University Institutional Review Board reviewed our study protocol and exempted the analyses from the need for approval. This study adhered to the tenets of the Declaration of Helsinki.

The AREDS was a prospective, multicenter, randomized, controlled trial aiming to evaluate the effects of oral supplements on AMD and the progression of cataracts. Previous AREDS reports described the study design extensively.^30^ In brief, 11 retinal specialty clinics recruited 4757 participants 55 to 80 years of age from 1992 to 1998. The AREDS research group took standard CFPs at the time of enrollment, at the 2-year follow-up visit, and annually after that.^30^ The University of Wisconsin fundus photograph reading center graded the presence of GA, AMD severity, and other AMD-related fundus abnormalities in all CFPs from the AREDS.^31^ Because the AREDS data files did not contain the delineations of GA lesions, we manually delineated GA and marked the foveal center on 1654 CFPs of 365 eyes7 using ImageJ 1.52p software (National Institutes of Health, Bethesda, MD, USA).^32^ We reported the methods of image grading in a previous paper.^7^ We excluded eyes with neovascular AMD at any visit from our study. We defined gradable GA as GA lesions that we were able to delineate the GA borders on CFPs.

The AREDS dataset contains two data files (AREDS 2010 and 2014 data files). The two data files contain high-quality CFPs from overlapping patients but were taken at different follow-up visits and were uploaded to dbGaP at year 2010 and 2014, respectively. Team A and B independently graded each CFP for GA, the foveal center, and the optic disc in the AREDS 2010 data file for evaluating the intergrader reproducibility.^7^ We calculated the Dice coefficient between GA delineations by the two teams. The Dice coefficient was defined as two times the intersection of two areas divided by the sum of the individual areas (a value close to 1 indicates perfect agreement).^33^ We also calculated the intraclass correlation coefficient (ICC) for GA size and growth rate measurements using the two-way model with single measurements.^34^

In the present study, we included all eyes that had visits with gradable GA spaced 1 year apart. For each eye that met the inclusion criteria, we included only the first two visits that were 1 year apart. We termed the first visit as the baseline visit and the second visit as the 1-year visit . Because the primary aim of the study was to investigate the expansion rate at the GA border, we excluded GA lesions delineated in only one of the two visits of an eye with multifocal GA. Based on our GA delineations, we extracted GA area, GA perimeter, GA circularity index, lesion number (multifocal GA was defined as ≥2 GA lesions in an eye), the presence of GA in the fellow eye, and distance from each point on the GA border to the foveal center. The GA circularity index was defined as (4 × π × total area)/total perimeter^2^.^20^ A GA circularity index of 1 would indicate a circular GA lesion. For the fellow eyes for which we were unable to segment GA lesions, we used the original AREDS gradings to determine the presence of any GA lesion.

### Quantification of GA Border Expansion Rates and Statistical Analysis

**Figure 1.**
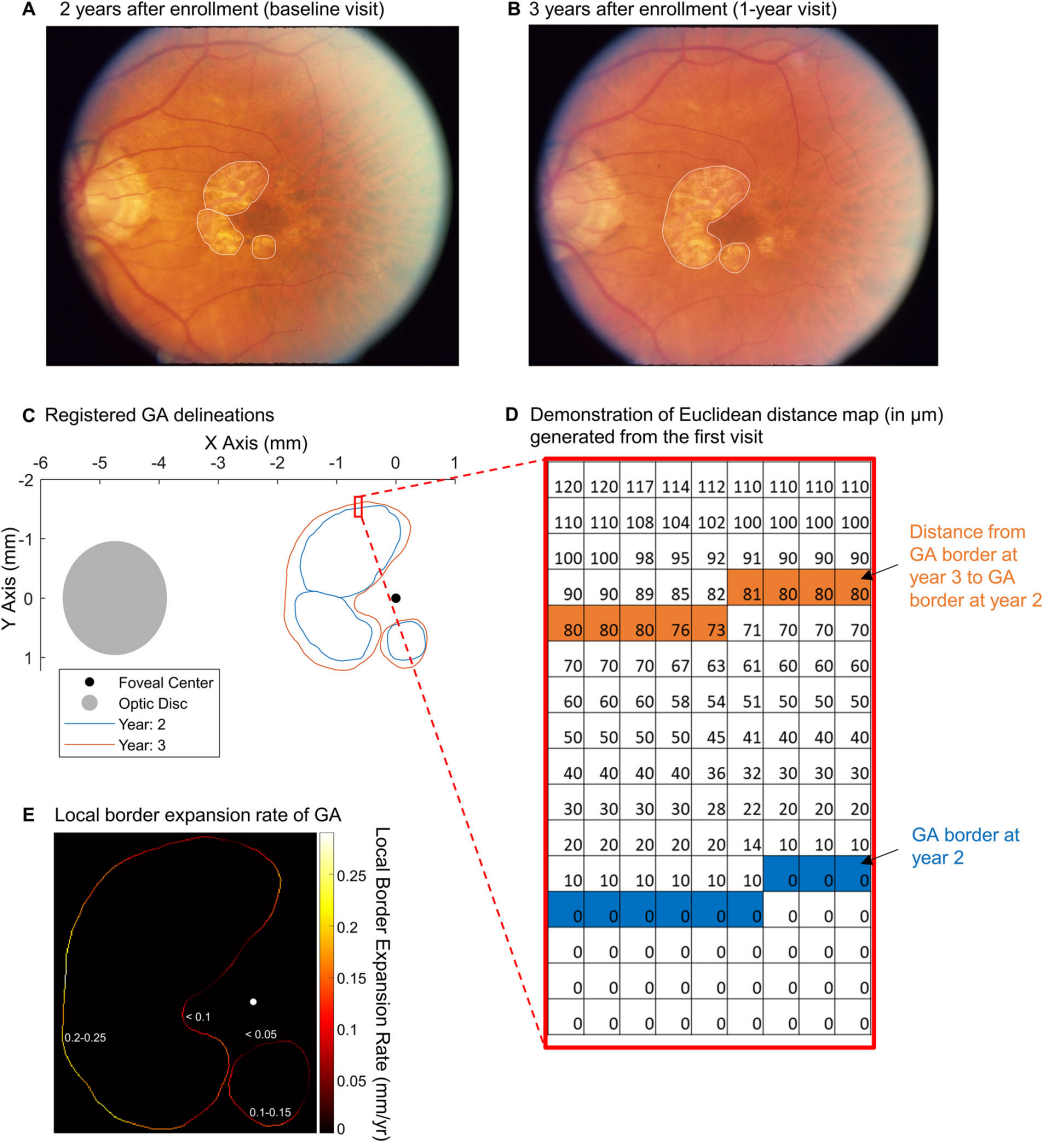
Demonstration of local GA BER. (A) Color fundus photograph of an eye 2 years after enrollment in the AREDS. We considered this visit to be the baseline visit of the eye in the present study. The *white lines* represent manually delineated GA borders. (B) The same eye 1 year later (i.e., 1-year visit). (C) Delineation of the GA border from the two visits was registered based on three points at vessel bifurcations and shown in a coordinate system centered at the fovea. (D) A matrix showing a part of the Euclidean distance map generated based on GA at the baseline visit (year 2 after enrollment). The *blue pixels* represent the GA border at the baseline visit. The value in each pixel (pixel width = 10 µm) of the Euclidean distance map indicates the shortest distance from this pixel to the GA at the baseline visit. *Orange pixels* represent the GA border at the 1-year visit. The value in each *orange pixel* represents the shortest distance from the GA border at the 1-year visit to the GA border at the baseline visit (i.e., local GA BER). (E) Based on the Euclidean distance map, we determined the local GA BERs (mm/y) at each pixel of the GA border at the 1-year visit (demonstrated in the heatmap). Note that the BERs were consistently below 0.1 mm/y along the GA border closer to the fovea and increased at distances farther from the fovea (see values marked on the heatmap). We calculated eye-specific BERs as the mean of local BERs of all pixels on the GA border in each eye over 1 year.

**Figure 2.**
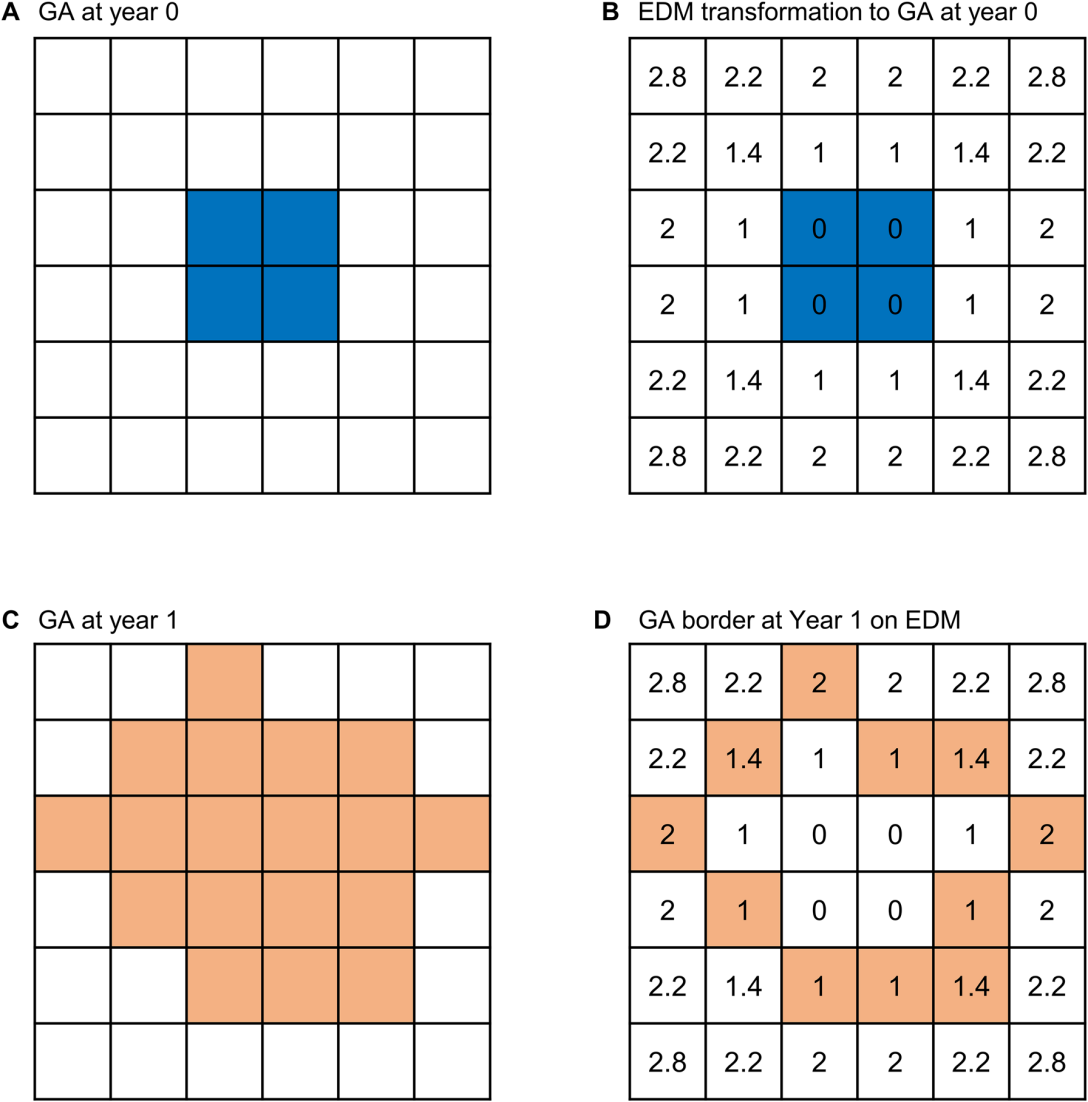
Illustration of GA BER measurement based on the Euclidean distance map (EDM). The *large square* represents the imaging field, and the *small squares* represent individual pixels. (A) A GA lesion affecting four pixels (*blue squares*) at year 0. (B) After we performed an EDM transformation to the mask of the GA lesion at year 0, the value in each pixel of the EDM now indicated the shortest distance from this pixel to the GA lesion. The values in pixels affected by GA (*blue pixels*) are 0. (C) The enlarged GA lesion (*orange squares*) after 1 year of follow-up. (D) After we overlay the border of GA lesion at year 1 on the EDM shown in (B), we were able to obtain the shortest distances (i.e., values in the *orange pixels*) from the pixels on the GA border at year 1 to the GA border at year 0. These values represent GA BERs over 1 year of follow-up.

We performed the statistical analysis in R 3.6.2 (R Foundation for Statistical Computing, Vienna, Austria) and MATLAB (The MathWorks, Natick, MA, USA). To quantify the linear expansion rate of the GA border, we registered GA border delineations from the baseline and 1-year visits of each eye in a virtual coordinate system based on three points at vessel bifurcations (from Figs. 1A and 1B to 1C).^7^ Then, we employed a Euclidean distance map to calculate the expansion rate at each point on the GA border, as proposed by Uji et al.^28^ We illustrate the process of calculating the GA BER based on the Euclidean distance map in Figure 2. In each eye, we applied a Euclidean distance map transformation to the mask of all GA lesions at the baseline visit so that every pixel beyond the baseline GA border was encoded with the shortest distance to the baseline GA border (demonstrated in Fig. 1D and Fig. 2B). We defined the pixel size as 10 µm in width because it was approximately the size of the imaging resolution of a color fundus camera and the diameter of a RPE cell.^35^^,^^36^ In eyes with multifocal GA, the unions of the lesions were taken prior to analysis. In other words, the pixels within at least one GA lesion border were deemed to have atrophy, and the pixels outside all GA lesion borders were deemed to have healthy cells. Then, we extracted the value in each pixel on the GA border at the 1-year visit. This value represented the local GA BER, equivalent to the shortest distance from a pixel on the GA border at the 1-year visit to the GA border at the baseline visit. Figure 1E shows a heatmap demonstrating the local GA BER at all pixels of the GA border at the 1-year visit in an eye.

To investigate the variation of GA location and local BER as a function of retinal eccentricity, we divided the macula into seven topographic regions with seven concentric circles centered at the fovea with a 0.5-mm increment in radius up to 3.5 mm away from the foveal center. We did not investigate regions beyond 3.5-mm retinal eccentricity because the radius of the fundus imaging field of view was 15° (approximately 4.3 mm) in the AREDS, and local GA BERs between 3.5 and 4 mm were highly variable due to poor imaging quality at the edge of imaging field (e.g., Fig. 1A) and the small number of eyes (31 eyes) with a GA border in this region. We performed linear mixed-effects modeling to investigate the association between GA BER as a function of retinal eccentricity after adjustment for baseline GA area, with a random intercept effect at the eye level and random intercept and slope effects at the patient level. Because a topographic region in an eye could contain more than one GA border pixel (i.e., more than one local BER), we calculated the mean local BER in each region for each eye and then calculated the mean and 95% confidence interval (CI) for each region. We did not calculate the GA BER in a topographic region if the region was not affected by GA. Next, we calculated the percentage of area affected by GA as GA area in each region divided by the total retinal area in the corresponding region at the baseline visit of each eye. We used a spline function in MATLAB to estimate the trend line for the percentage of area affected by GA as a function of retinal eccentricity. We performed the above analyses again after we used concentric circles to redivide the macula into seven regions having the same area: (π × 3.5^2^)/7 = 5.50 mm^2^.

Because the local GA BER was defined as the shortest distance from a pixel on the GA border at year 1 to the GA border at year 0, we could not quantify local GA BERs reliably in regions where lesion margins merged or progressed along nonlinear paths (Supplementary Fig. S1). In the first case, GA margins disappeared after merging at year 1 so the local GA BERs could not be calculated in this region (Supplementary Figs. S1A, S1B). In the second scenario, measurement of the GA BERs in some pixels had to pass through a non-atrophic region, which would not reflect the actual GA progression rate (Supplementary Figs. S1C, S1D). Therefore, we manually reviewed all GA lesions and reanalyzed the GA BERs as a function of retinal eccentricity after removing eyes with GA margins that merged or had nonlinear growth. We deemed an eye as having GA margins merging if the number of GA lesions in the eye decreased or if different GA margin segments of the same lesion merged into one another over the 1-year follow-up. Eyes with nonlinear GA growth were identified if the shortest path from a GA margin segment at the baseline visit to year 1 visit passed through a non-atrophic region.

We next investigated the variation of GA area and local BER in four different quadrants within the Early Treatment Diabetic Retinopathy Study (ETDRS) grid. The center circle radius was 0.5 mm, and the outer circle radius was 3 mm. We performed the analyses by quadrants because the density of some retinal cells (e.g., S-cones^37^) and structures (e.g., choroidal thickness)^38^ may differ by quadrants, despite the radial symmetry of many retinal neurons and supporting cells around the fovea.^37^^,^^39^ Also, the sunlight may affect the inferior quadrant more than the superior quadrant. Similar to above, we calculated the percentage of area affected by GA as the GA area in each quadrant divided by total retinal area in the corresponding quadrant at the = baseline visit of each eye. Among eyes with GA involving all four quadrants, we calculated the mean local GA BER in each quadrant of the ETDRS grid. We compared the percentage of area affected by GA and the local GA BERs across different quadrants using the Friedman test.^7^

We calculated the eye-specific BER as the mean of the local BERs of all pixels on the GA border for each eye. We then employed a univariable linear mixed-effects model (with the eye as the unit of analysis and random intercept effects for different patients) to investigate the relationship between eye-specific BER and the factors listed in the Table. Because the GA border location changes over time in each eye, we defined the distance between the GA border and the foveal center as the mean distance from all pixels on the GA border to the foveal center averaged between the baseline and 1-year visits. We repeated the same analysis by using the GA area growth rate as the outcome measure. We performed a linear mixed-effects model with the eye as the unit of analysis to investigate the association between the eye-specific BER and GA perimeter-adjusted growth rate (defined as GA area growth rate divided by mean GA perimeter between the two follow-up visits).^23^

**Table.** Univariable Linear Mixed-Effects Model of Geographic Atrophy Growth Rate

	GA Area Growth Rate (mm^2^/y)		Eye-Specific GA Border Expansion Rate (mm/y)^*^	
	Estimate (95% CI)	*P*	Estimate (95% CI)	*P*
Baseline GA area (mm^2^)	0.17 (0.14–0.19)	<0.001	0.002 (−0.000 to 0.004)	0.051
Baseline GA perimeter (mm)	0.12 (0.11–0.14)	<0.001	0.001 (−0.000 to 0.002)	0.15
Baseline lesion number	0.25 (0.10–0.39)	<0.001	−0.002 (−0.010 to 0.006)	0.66
Baseline multifocal GA	1.06 (0.56–1.55)	<0.001	0.014 (−0.014 to 0.042)	0.34
Baseline GA in the fellow eye	0.78 (0.28–1.29)	0.002	−0.005 (−0.032 to 0.023)	0.73
Baseline circularity index^†^	−2.81 (−3.57 to −2.04)	<0.001	−0.031 (−0.077 to 0.015)	0.18
Distance between GA border and the foveal center (mm)^‡^	1.87 (1.57–2.17)	<0.001	0.037 (0.016–0.058)	<0.001

* Eye-specific GA border expansion rates were calculated as the mean local border expansion rates of all points on the GA border in each eye over 1 year.

† The circularity index was calculated as (4 × π × total area)/Total perimeter^2^, ranging from 0 to 1. A circularity close to 1 indicates a circular GA, and a circularity index close to 0 indicates a noncircular or multifocal GA.

‡ The distance between the GA border and the foveal center was defined as the mean distance from all pixels on the GA border to the foveal center averaged between the two visits spaced over 1 year.

## Results

### Patient Characteristics and Intergrader Reproducibility

We included 237 eyes from 160 patients (55% were females) who had gradable GA followed for 1 year. At baseline (i.e., the first of the two visits), the mean ± SD age of patients was 70.5 ± 5.3 years, total GA area was 5.8 ± 7.7 mm^2^, total GA perimeter was 11.7 ± 10.7 mm, the circularity index was 0.61 ± 0.30, the lesion number was 1.7 ± 1.7, and the mean distance between the GA border and the foveal center was 1.3 ± 0.6 mm (Supplementary Table S1).

Teams A and B independently graded GA in 240 visits (54 eyes) for the intergrader reproducibility test. We found excellent intergrader reproducibility of GA area (ICC = 0.997),^23^ GA perimeter (ICC = 0.98),^23^ GA perimeter-adjusted growth rate (ICC = 0.95),^23^ and mean distance from the GA border to the foveal center (ICC = 0.98) (Supplementary Fig. S2A). The mean ± SD Dice coefficient of GA delineations by the two teams was 0.82 ± 0.13. Among the 54 eyes, 27 eyes had gradable GA followed for 1 year. We calculated eye-specific GA BERs in these eyes based on gradings by each team. The eye-specific GA BERs measured by the two teams had a mean difference of −0.01 mm/y and an ICC of 0.64. The ICC increased to 0.81 after we removed one outlier from the analysis (Supplementary Fig. S2B). The outlier is shown in Supplementary Figure S3, in which the GA margins are unclear and the two grader teams disagreed significantly on the GA border delineations in the second visit. The median distance between the foveal center markings by the two teams was 199 µm (IQR = 187),^23^ comparable to the median difference (178 µm) reported by Sunness et al.^40^

### Variation of GA Growth Rate and Prevalence by Retinal Eccentricity

**Figure 3.**
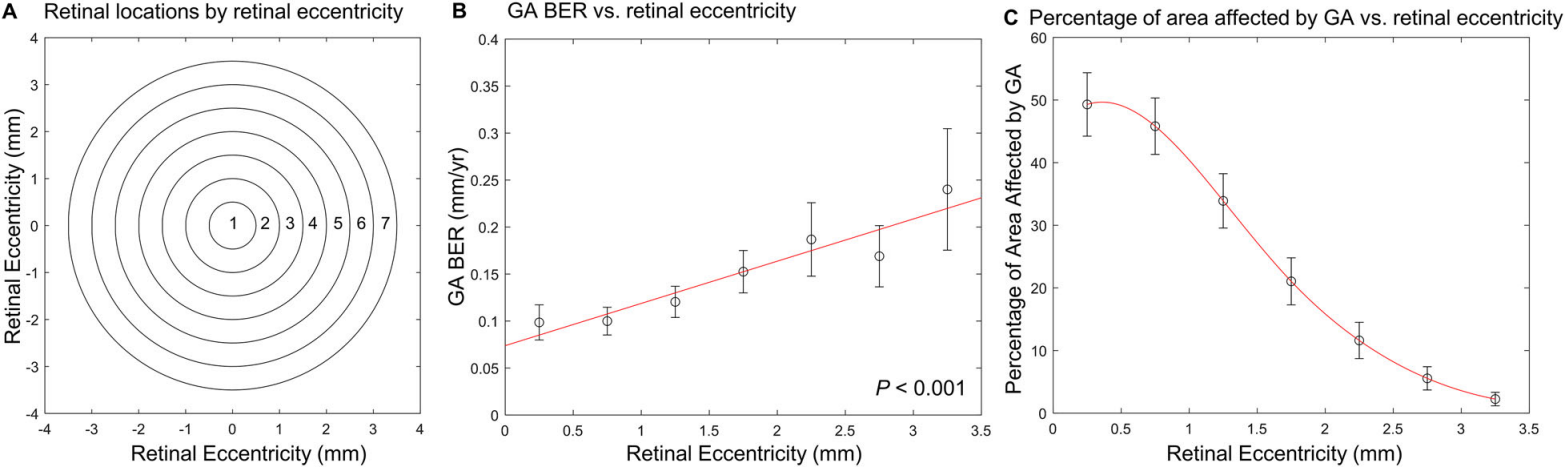
Variation of GA progression and distribution as a function of retinal eccentricities (*N* = 237 eyes). The *error bar* represents 95% CIs for the mean. The *red line* represents the trendline. (A) We divided the retina into seven topographic zones using concentric circles (centered at the fovea) with a 0.5-mm increment in radius (up to 3.5 mm). (B) Local GA BERs increased as a function of retinal eccentricity (*P* < 0.001). The GA BER was 0.10 mm/y (95% CI, 0.08–0.12; *n* = 149 eyes) in zone 1 and increased by 2.4-fold to 0.24 mm/y (95% CI, 0.18–0.31; *n* = 54 eyes) in zone 7. (C) We calculated the percentage of retinal area affected by GA as the GA area in each zone divided by the total retinal area in the corresponding zone. As the retinal eccentricity increased from 0 mm to 3.5 mm, the percentage of retinal area affected by GA decreased from 49.3% to 2.3%. On average, GA affected approximately 50% of the retina within 1 mm from the foveal center; by comparison, GA affected less than 15% of the retina beyond 2-mm retinal eccentricity.

We quantitatively measured local GA BERs in zones across seven retinal eccentricities (Fig. 3A). Local GA BERs increased progressively as a function of retinal eccentricity (*P* < 0.001) after adjustment for baseline GA area. Local GA BER was 0.10 mm/y (95% CI = 0.08–0.12; *n* = 149 eyes) at 0- to 0.5-mm retinal eccentricity and increased by 2.4-fold to 0.24 mm/y (95% CI, 0.18–0.31; *n* = 54 eyes) at 3.0- to 3.5-mm retinal eccentricity (Fig. 3B). Interestingly, the percentage of area affected by GA followed the opposite trend (Fig. 3C). As the retinal eccentricity increased from 0 mm to 3.5 mm, the percentage of area affected by GA decreased from 49.3% to 2.3% (Fig. 3C). On average, GA affected approximately 50% of the retina within 1 mm from the foveal center and affected less than 15% of the retina beyond 2-mm retinal eccentricity. After we removed GA lesions that had any GA margins merging (66 eyes) or nonlinear growth (eight eyes), the local GA BERs increased progressively as a function of retinal eccentricity (*P* < 0.001) (Supplementary Fig. S4).

The results were consistent after we redefined the seven topographic zones as zones with the same areas (5.5 mm^2^) but different widths (Supplementary Fig. S5A). The local GA BERs still increased as a function of retinal eccentricity (*P* < 0.001) (Supplementary Fig. S5B), whereas the percentage of retinal area affected by GA decreased progressively (Supplementary Fig. S5C).

### Variation of GA Growth Rate and Prevalence in Different Quadrants

**Figure 4.**
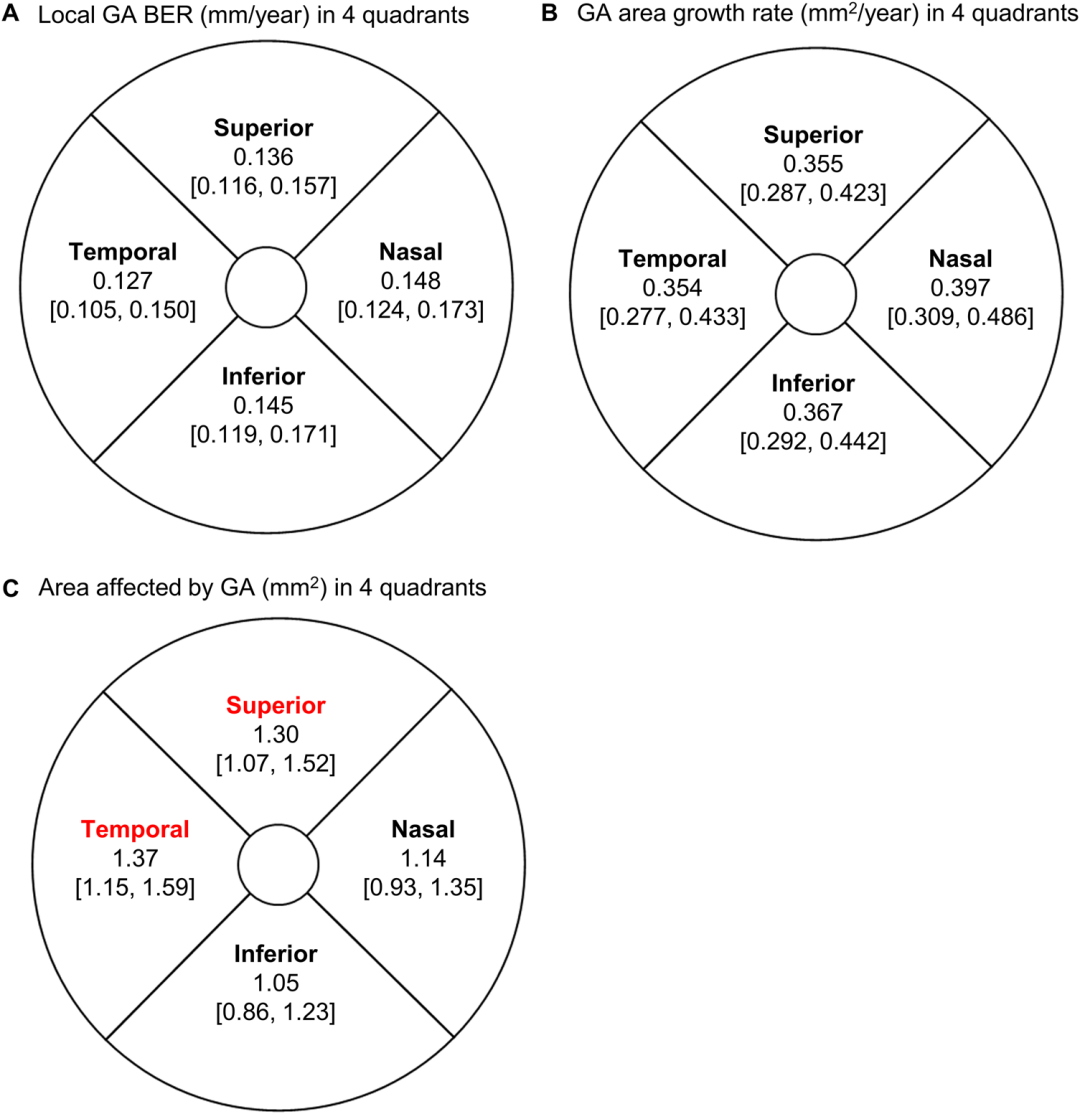
Variation of the growth rate of GA and area affected by GA in four quadrants within the ETDRS grid. The radius of the center circle was 0.5 mm, and the radius of the outer circle was 3 mm. (A) We determined the local GA BERs in each quadrant of the ETDRS grid among eyes with GA involving all four quadrants (*n* = 137 eyes). Local GA BERs did not differ significantly across the four quadrants based on the Friedman test (*P* = 0.23). (B) Similarly, the growth rate of GA area (*n* = 137 eyes) did not differ significantly across the four quadrants based on the Friedman test (*P* = 0.27). (C) At the baseline visit of all included eyes (237 eyes), the GA area was significantly different across the four quadrants of the ETDRS grid based on the Friedman test (*P* = 0.002). The GA area was significantly higher in the temporal than in the nasal quadrant (*P* = 0.02) and was significantly higher in the superior than in the inferior quadrant (*P* = 0.01).

The local GA BERs were comparable across four quadrants of the ETDRS grid (range, 0.127–0.148 mm/y; *P* = 0.23; *n* = 137 eyes) (Fig. 4A). Similarly, the GA area growth rate was comparable across four quadrants (range, 0.354–0.397 mm^2^/y; *P* = 0.27; *n* = 137 eyes) (Fig. 4B). Interestingly, the GA areas were significantly different across the four quadrants (*P* = 0.002; *n* = 237 eyes) (Fig. 4C). The GA area was higher in the temporal than in the nasal quadrant (*P* = 0.02) and was higher in the superior than in the inferior quadrant (*P* = 0.01), consistent with our previous report in a larger cohort (365 eyes).^7^ We reported a pairwise comparison of GA area across four quadrants in that previous paper.

### Eye-Specific GA Border Expansion Rates Were Uncorrelated with Baseline GA Morphology

**Figure 5.**
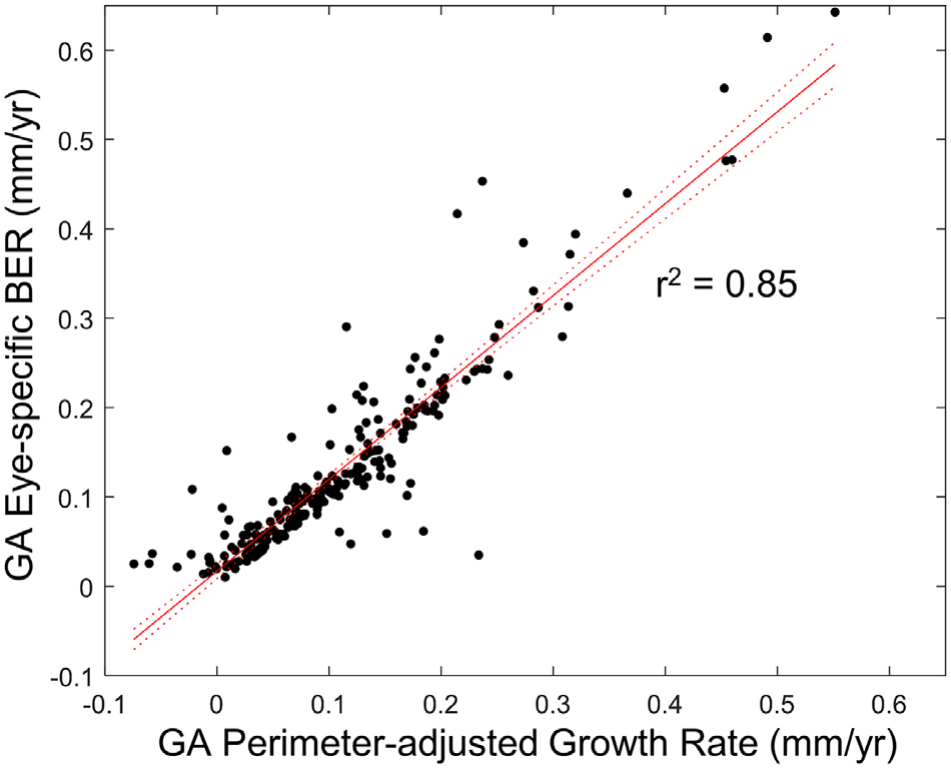
Eye-specific GA BERs were strongly associated with GA perimeter-adjusted growth rates. Both parameters were calculated based on visits spaced over 1 year in 237 eyes. The eye-specific BER was calculated as the mean local BERs of all pixels on the GA border over 1 year using the Euclidean distance map (Fig. 1). We calculated the GA perimeter-adjusted growth rate as (Total GA area at year 1 – total GA area at year 0)/(Mean GA perimeter between first and second visits). Both parameters reflect the mean expansion rate of the leading edge of GA (Supplementary Fig. S6), although they were measured differently. The strong association between the two parameters (*r*^2^ = 0.85; *P* < 0.001) indicates that the two parameters are equivalent for eyes with a relatively short follow-up duration (e.g., 1 year).

The Table shows that the GA area growth rate was associated with baseline GA area (*P* < 0.001), lesion perimeter (*P* < 0.001), lesion number (*P* < 0.001), lesion focality (*P* < 0.001), GA in the fellow eye (*P* = 0.002), and GA circularity index (*P* < 0.001). In contrast, using eye-specific GA BERs as the outcome measure eliminated the significant associations with these factors (*P* = 0.051–0.73) (Table). Both GA area growth rate and eye-specific GA BER were significantly correlated with the distance between the GA border and the foveal center (*P* < 0.001). Additionally, eye-specific GA BER was strongly associated with our previously proposed GA perimeter-adjusted growth rate23 in the same cohort (*r*^2^ = 0.85; *P* < 0.001) (Fig. 5).

## Discussion

Previous natural history studies of GA focused on the growth rate of the total GA area.^14^^,^^16^ A few recent studies have investigated the variation of local GA progression kinetics in centrifugal and centripetal directions,^5^ in clockwise directions,^28^^,^^41^ and within ETDRS subfields.^42^^,^^43^ Most of these studies had fewer than 100 eyes and did not quantify GA progression across multiple retinal eccentricities. A priori, it is unclear if the linear BER of GA stays relatively constant or varies progressively across different retinal eccentricities. Herein, we employed the Euclidean distance map to quantify local GA BERs as a function of retinal eccentricity in 237 eyes from 160 patients in the AREDS. To our knowledge, this study is the first patient-level analysis that has demonstrated a progressive increase of local GA BERs from the foveal center to 3.5-mm retinal eccentricity, with a 2.4-fold difference between the maximum and minimum growth rate. Interestingly, the percentage of area affected by GA followed the opposite trend, as it decreased from 49.3% to 2.3% and the retinal eccentricity increased from 0 to 3.5 mm. The GA area growth rates and local GA BERs were comparable across four macular quadrants, but the area of GA lesions was statistically significantly higher in the temporal and superior quadrants than in the nasal and inferior macular quadrants, consistent with our prior results.^7^ The overall macular GA area growth rate (mm^2^/y) was significantly associated with baseline GA area, GA perimeter, lesion number, lesion focality, GA in the fellow eye, and circularity index; in contrast, eye-specific GA BER (mm/y) was not correlated with any of these prognostic factors. Both GA growth rate measurements were positively associated with distance from the GA border to the foveal center.

The finding of a progressive increase in local GA BERs as the retinal eccentricity increased from 0 to 3.5 mm is consistent with the foveal sparing phenomenon^5^^–^^8^ and the previous observation of higher GA growth rates in extrafoveal regions than in the foveal regions.^5^^,^^42^^,^^43^ Our previous meta-analysis of study-level data also suggested a similar, progressive increase of the GA growth rate from 0- to 3.6-mm retinal eccentricity.^27^ Additionally, the present study demonstrated comparable GA BERs across four macular quadrants, consistent with the report by Uji et al.^28^ Of note, our previous meta-analysis suggested a lower GA growth rate beyond 3.6-mm retinal eccentricity,^27^ which was not assessed in the present study due to the limited imaging field of view in the AREDS, poor imaging quality at the imaging margin, and few data points outside 3.5-mm retinal eccentricity. Future studies using widefield imaging are required to investigate GA growth rates beyond this region.

We have previously demonstrated that the GA perimeter-adjusted growth rate is not correlated with baseline GA area, lesion number, or circularity index, implying that the linear expansion rate of the leading edge of GA is independent of lesion morphology.^23^ In the present study, we validated this hypothesis by directly measuring the linear expansion rate of the GA border (i.e., local GA BERs) and correlating it with baseline morphological factors (Table). Importantly, both the eye-specific GA BER and GA perimeter-adjusted growth rate reflect the mean expansion rate of the leading edge of GA (Supplementary Fig. S6), although they are measured differently. Under the ideal circumstance (i.e., precisely delineated GA border and infinitely small follow-up duration), the two parameters should have the same values. But in reality, the GA perimeter-adjusted growth rate is influenced by measurement errors in GA area and perimeter, whereas eye-specific GA BER is affected by measurement errors in the linear distance determined from the Euclidean distance map. For example, Supplementary Figure S7 demonstrates a case where eye-specific GA BERs differed from GA perimeter-adjusted growth rates. In this eye, the eye-specific GA BER was 0.29 mm/y but the GA perimeter-adjusted growth rate was only 0.12 mm/y. The difference occurred when GA delineation at the follow-up visit had a regional GA border protrusion, which led to a large mean linear distance measurement of GA border expansion, although the change in GA area measurement was relatively small. The GA border protrusion could occur due to actual GA growth at the border, the onset of new GA lesion near the GA margin, or measurement error. Nonetheless, the strong association between eye-specific GA BERs and GA perimeter-adjusted growth rates (*r*^2^ = 0.85; *P* < 0.001) (Fig. 5) indicates that the two parameters are equivalent for most eyes within a relatively short time frame (e.g., 1 year). Compared with eye-specific GA BERs, the GA perimeter-adjusted growth rate is simpler to quantify, as graders would only have to measure the GA area and perimeter, and it has higher intergrader reproducibility (ICC = 0.95 vs. 0.64). Therefore, GA perimeter-adjusted growth rates may be preferred over eye-specific GA BERs in studies aiming to evaluate the overall macular GA progression rate.

In principle, quantification of the local GA BERs (i.e., the linear expansion rate at the local GA border) may be crucial to some clinical trials and natural history studies, particularly for trials designed to measure the efficacy of regional treatment (e.g., subretinal stem cell transplantation) on local GA progression rate.^41^ The local GA BERs can also serve as an outcome measure for studies aiming to associate regional microanatomy (e.g., choriocapillaris flow deficit, local hyperreflective foci) with local GA progression kinetics.^44^^,^^45^ Future studies can use the same method to explore the applications of local BER in inherited retinal degenerations, which may include evaluating the impact of gene therapy via subretinal injection on local atrophy progression.

The underlying mechanism responsible for the differential GA progression kinetics in the macula is unclear, but several hypotheses have been proposed. By examining these hypotheses based on our observed topographic variation of GA progression kinetics, we may gain insight into GA expansion mechanisms. One hypothesis is that the rod photoreceptors are more vulnerable to atrophy progression than cones.^5^^,^^27^ Some histological studies have shown that the rod loss was disproportionally higher than the cone loss in aging in various AMD stages.^39^^,^^46^^–^^48^ Functional studies also demonstrated that the loss of rod-mediated scotopic sensitivity was greater than the loss of cone-mediated photopic sensitivity in AMD patients.^49^^,^^50^ A recent study^44^ suggested that the slowing of rod-mediated dark adaptation in early AMD peaked at the fovea rather than the perifovea or periphery, corresponding to our finding regarding the topographic variation of the percentage of retinal area affected by GA. Interestingly, our observed GA BERs followed a topographic change similar to that of the rod density and rod-to-cone ratio, which increased progressively from the foveal center to 3.5 mm eccentricity.^39^ We hypothesize that local GA progression kinetics are higher in regions with higher rod density, which are more susceptible to atrophy. This hypothesis is supported by a recent study by Pfau et al.,^51^ who demonstrated that rod dysfunction temporally precedes cone retinal sensitivity impairment near the GA border. Future studies should continue to investigate the role of the rod system in GA expansion and whether rods are a viable therapeutic target for slowing GA progression.^24^

Another possible mechanism responsible for the topographic variation in GA progression rates is the distribution of macular pigment (lutein, zeaxanthin, and meso-zeaxanthin), which possesses antioxidant properties.^52^ Recent studies showed that the density of macular pigment drops sharply from the foveal center to the perifovea,^53^^,^^54^ in an inverse relationship of the topographic variation of GA progression rate.

A third hypothesis is that a higher choriocapillaris flow deficit correlates with faster GA expansion. However, this hypothesis does not explain our observation because choriocapillaris flow deficits peak at the fovea in the elderly,^55^^–^^57^ but our observed local GA BERs peaked at 3.5-mm retinal eccentricity. Additionally, the progressive increase of GA BER as a function of retinal eccentricity does not appear to correlate with the topographic variation of the S-cone density,^37^ ganglion cell density,^58^ superficial capillary plexus density,^76^ or the deep capillary plexus.^59^ RPE density dropped approximately 10% from the foveal center to 3.5-mm retinal eccentricity, consistent with the general trend of the topographic variation of GA BERs.^59^ However, the slight difference in RPE density alone may not explain the 2.4-fold increase in GA BERs unless RPE cells at different retinal eccentricities have significant structural or functional differences^60^^,^^61^ that may influence GA progression.

An important finding in our study is that the topographic variation of the GA BERs is in the opposite direction of the variation in the percentage of area affected by GA. As noted above, topographic variation of the GA BER appears to correlate with anatomical changes in the rod density; in contrast, the topographic variation of GA distribution follows a similar trend as the cone density.^7^^,^^62^ This topographic difference in GA expansion rate and GA distribution suggests that GA onset may be driven by different biological processes from GA expansion. Previous histological studies have suggested that GA lesions typically begin over drusen,^63^^–^^65^ consistent with the similar topographic variation between the soft drusen height53 and the percentage area affected by GA in our study. In comparison, GA expansion is unlikely to be driven by drusen, as the atrophic lesions are commonly not surrounded by drusen. Additional evidence for the distinct biological processes between GA initiation and expansion is that multiple factors associated with the presence of GA are not associated with higher GA growth rates. For example, the complement factor H genotype is strongly associated with the incidence of GA,^66^^–^^68^ but it is not significantly associated with GA progression rates based on several studies.^18^^,^^69^^,^^70^ Future studies should consider GA incidence and expansion as two separate biological processes and select the therapeutic target based on whether they aim to prevent GA onset or slow GA expansion.

Our study has several limitations. First, although we used data from a prospective study, the analysis was retrospectively designed. Second, because the AREDS dataset contains only CFPs, we determined the foveal center based on the CFPs. This technique is imprecise and resulted in a 199-µm median distance between the foveal center markings by the two teams in our study, similar to what has been reported by Sunness et al.^40^ Due to this limitation, we employed a relatively large retinal eccentricity interval (500 µm) to quantify the topographic variation of local GA BERs and GA distributions. Future studies can use optical coherence tomography (OCT) or OCT angiography to identify the foveal center more accurately. Third, we were not able to assess GA BERs outside 3.5 retinal eccentricity. Fourth, there are some measurement errors in delineating the GA border that may contribute to the relatively low intergrader reproducibility of eye-specific GA BERs (ICC = 0.64, which improves to 0.81 after removing one outlier). Future studies may use fundus autofluorescence (FAF) or OCT to delineate GA borders more precisely. Notably, several previous studies have shown that overall macular GA growth rates measured on CFP, FAF, and OCT are comparable,^19^^,^^22^^,^^71^^–^^73^ but it is still unknown if GA BER measurements differ in different imaging modalities. Fifth, the local GA BER is an objective measure to quantify the linear expansion rate of the GA border over time, but it is not a reliable measure for regions where lesion margins merge or progress along nonlinear paths. The overall impact of these two cases on our findings may be relatively small, as evidenced by the similar GA BERs as a function of retinal eccentricity before and after removing GA lesions that had any GA margins merging or nonlinear growth (Fig. 3B vs. Supplementary Fig. S4). However, GA margins merging and nonlinear growth in some cases can be difficult for the graders to visually identify. Due to the lack of established or objective methods in detecting these cases, we had to employ subjective criteria to identify these lesions and could not rule out the possibility of misclassifying less obvious cases. Sixth, the local GA BERs in adjacent topographic zones in a given eye were likely correlated. The non-independent nature of local GA BERs might limit the accuracy of calculating *P* values for the association between GA BERs and retinal eccentricity. Finally, due to the lack of FAF, OCT, and OCT angiography images, we were not able to investigate the association of GA BERs with retinal microstructure and several previously proposed biomarkers, including hyperfluorescence signals,^74^^,^^75^ hyperreflective foci,^45^ and choriocapillaris flow deficits.^44^

In conclusion, we quantified the topographic variation of GA progression kinetics in 237 eyes from 160 patients in the AREDS. To our knowledge, this study is the first to demonstrate a progressive increase of the local GA BER from the foveal center to 3.5-mm retinal eccentricity. The topographic variation of GA BERs is in the opposite direction of the variations in the percentage of area affected by GA, which may suggest that the expansion of existing GA lesions and the onset of new lesions are mediated by different biological processes. The linear expansion rate of the GA border is not correlated with the baseline GA morphological factors, consistent with our previous report.^23^ These findings may shed light on the underlying mechanism for GA initiation and the mechanism for GA expansion and may assist the design of future clinical trials.

## Supplementary Material

Supplement 1

Supplement 2

Supplement 3

Supplement 4

Supplement 5

Supplement 6

Supplement 7

Supplement 8

## References

[1] Wong WL, Su X, Li X, et al. Global prevalence of age-related macular degeneration and disease burden projection for 2020 and 2040: a systematic review and meta-analysis. *Lancet Glob Health*. 2014; 2: e106–e116. 25104651 10.1016/S2214-109X(13)70145-1

[2] Thiele S, Pfau M, Larsen PP, Fleckenstein M, Holz FG, Schmitz-Valckenberg S. Multimodal imaging patterns for development of central atrophy secondary to age-related macular degeneration. *Invest Ophthalmol Vis Sci*. 2018; 59: AMD1–AMD11. 29558532 10.1167/iovs.17-23315

[3] Li M, Huisingh C, Messinger J, et al. Histology of geographic atrophy secondary to age-related macular degeneration: a multilayer approach. *Retina*. 2018; 38: 1937–1953. 29746415 10.1097/IAE.0000000000002182 PMC6166696

[4] Sunness JS, Gonzalez-Baron J, Applegate CA, et al. Enlargement of atrophy and visual acuity loss in the geographic atrophy form of age-related macular degeneration. *Ophthalmology*. 1999; 106: 1768–1779. 10485549 10.1016/S0161-6420(99)90340-8

[5] Lindner M, Boker A, Mauschitz MM, et al. Directional kinetics of geographic atrophy progression in age-related macular degeneration with foveal sparing. *Ophthalmology*. 2015; 122: 1356–1365. 25972258 10.1016/j.ophtha.2015.03.027

[6] Sunness JS, Rubin GS, Zuckerbrod A, Applegate CA. Foveal-sparing scotomas in advanced dry age-related macular degeneration. *J Vis Impair Blind*. 2008; 102: 600–610. 20224750 PMC2836024

[7] Shen LL, Sun M, Ahluwalia A, et al. Relationship of topographic distribution of geographic atrophy to visual acuity in nonexudative age-related macular degeneration. *Ophthalmol Retina*. 2021; 5: 761–774. 33212271 10.1016/j.oret.2020.11.003 PMC12807493

[8] Shen LL, Sun M, Ahluwalia A, et al. Natural history of central sparing in geographic atrophy secondary to non-exudative age-related macular degeneration [published online ahead of print]. *Br J Ophthalmol*. 2020, 10.1136/bjophthalmol-2020-317636. PMC8813644 33361441

[9] Veritti D, Sarao V, Samassa F, et al. State-of-the art pharmacotherapy for non-neovascular age-related macular degeneration. *Expert Opin Pharmacother*. 2020; 21: 773–784. 32153203 10.1080/14656566.2020.1736557

[10] Kassa E, Ciulla TA, Hussain RM, Dugel PU. Complement inhibition as a therapeutic strategy in retinal disorders. *Expert Opin Biol Ther*. 2019; 19: 335–342. 30686077 10.1080/14712598.2019.1575358

[11] Liao DS, Grossi FV, El Mehdi D, et al. Complement C3 inhibitor pegcetacoplan for geographic atrophy secondary to age-related macular degeneration: a randomized phase 2 trial. *Ophthalmology*. 2020; 127: 186–195. 31474439 10.1016/j.ophtha.2019.07.011

[12] Jaffe GJ, Westby K, Csaky KG, et al. C5 inhibitor avacincaptad pegol for geographic atrophy due to age-related macular degeneration: a randomized pivotal phase 2/3 trial. *Ophthalmology*. 2021; 128: 576–586. 32882310 10.1016/j.ophtha.2020.08.027

[13] Sadda SR, Chakravarthy U, Birch DG, Staurenghi G, Henry EC, Brittain C. Clinical endpoints for the study of geographic atrophy secondary to age-related macular degeneration. *Retina*. 2016; 36: 1806–1822. 27652913 10.1097/IAE.0000000000001283 PMC5384792

[14] Shen L, Liu F, Grossetta Nardini H, Del Priore LV. Natural history of geographic atrophy in untreated eyes with nonexudative age-related macular degeneration: a systematic review and meta-analysis. *Ophthalmol Retina*. 2018; 2: 914–921. 31047226 10.1016/j.oret.2018.01.019

[15] Batioglu F, Gedik Oguz Y, Demirel S, Ozmert E. Geographic atrophy progression in eyes with age-related macular degeneration: role of fundus autofluorescence patterns, fellow eye and baseline atrophy area. *Ophthalmic Res*. 2014; 52: 53–59. 24993093 10.1159/000361077

[16] Fleckenstein M, Mitchell P, Freund KB, et al. The progression of geographic atrophy secondary to age-related macular degeneration. *Ophthalmology*. 2018; 125: 369–390. 29110945 10.1016/j.ophtha.2017.08.038

[17] Yehoshua Z, Rosenfeld PJ, Gregori G, et al. Progression of geographic atrophy in age-related macular degeneration imaged with spectral domain optical coherence tomography. *Ophthalmology*. 2011; 118: 679–686. 21035861 10.1016/j.ophtha.2010.08.018 PMC3070862

[18] Keenan TD, Agron E, Domalpally A, et al. Progression of geographic atrophy in age-related macular degeneration: AREDS2 report number 16. *Ophthalmology*. 2018; 125: 1913–1928. 30060980 10.1016/j.ophtha.2018.05.028 PMC6246813

[19] Schmitz-Valckenberg S, Sahel JA, Danis R, et al. Natural history of geographic atrophy progression secondary to age-related macular degeneration (Geographic Atrophy Progression Study). *Ophthalmology*. 2016; 123: 361–368. 26545317 10.1016/j.ophtha.2015.09.036

[20] Domalpally A, Danis RP, White J, et al. Circularity index as a risk factor for progression of geographic atrophy. *Ophthalmology*. 2013; 120: 2666–2671. 24206616 10.1016/j.ophtha.2013.07.047

[21] Pfau M, Lindner M, Goerdt L, et al. Prognostic value of shape-descriptive factors for the progression of geographic atrophy secondary to age-related macular degeneration. *Retina*. 2019; 39: 1527–1540. 29781974 10.1097/IAE.0000000000002206

[22] Shen LL, Sun M, Grossetta Nardini HK, Del Priore LV. Progression of unifocal vs. multifocal geographic atrophy in age-related macular degeneration: a systematic review and meta-analysis. *Ophthalmol Retina*. 2020; 4: 899–910. 32423772 10.1016/j.oret.2020.03.020 PMC7483721

[23] Shen LL, Sun M, Ahluwalia A, Young BK, Park MM, Del Priore LV. Geographic atrophy growth is strongly related to lesion perimeter: unifying effects of lesion area, number, and circularity on growth. *Ophthalmology Retina*. 2021; 5: 868–878. 33307218 10.1016/j.oret.2020.12.002

[24] Rosenfeld PJ, Dugel PU, Holz FG, et al. Emixustat hydrochloride for geographic atrophy secondary to age-related macular degeneration: a randomized clinical trial. *Ophthalmology*. 2018; 125: 1556–1567. 29716784 10.1016/j.ophtha.2018.03.059

[25] Holz FG, Sadda SR, Busbee B, et al. Efficacy and safety of lampalizumab for geographic atrophy due to age-related macular degeneration: Chroma and Spectri phase 3 randomized clinical trials. *JAMA Ophthalmol*. 2018; 136: 666–677. 29801123 10.1001/jamaophthalmol.2018.1544 PMC6145777

[26] Mones J, Biarnes M. The rate of progression of geographic atrophy decreases with increasing baseline lesion size even after the square root transformation. *Transl Vis Sci Technol*. 2018; 7: 40. 30619660 10.1167/tvst.7.6.40 PMC6314221

[27] Shen LL, Sun M, Khetpal S, Grossetta Nardini HK, Del Priore LV. Topographic variation of the growth rate of geographic atrophy in nonexudative age-related macular degeneration: a systematic review and meta-analysis. *Invest Ophthalmol Vis Sci*. 2020; 61: 2. 10.1167/iovs.61.1.2 PMC7205189 31995152

[28] Uji A, Nittala MG, Hariri A, Velaga SB, Sadda SR. Directional kinetics analysis of the progression of geographic atrophy. *Graefes Arch Clin Exp Ophthalmol*. 2019; 257: 1679–1685. 31147841 10.1007/s00417-019-04368-1

[29] Mailman MD, Feolo M, Jin Y, et al. The NCBI dbGaP database of genotypes and phenotypes. *Nat Genet*. 2007; 39: 1181–1186. 17898773 10.1038/ng1007-1181 PMC2031016

[30] Age-Related Eye Disease Study Research Group. The Age-Related Eye Disease Study (AREDS): design implications. AREDS report no. 1. *Control Clin Trials*. 1999; 20: 573–600. 10588299 10.1016/s0197-2456(99)00031-8 PMC1473211

[31] Age-Related Eye Disease Study Research Group. The Age-Related Eye Disease Study system for classifying age-related macular degeneration from stereoscopic color fundus photographs: the Age-Related Eye Disease Study report number 6. *Am J Ophthalmol*. 2001; 132: 668–681. 11704028 10.1016/s0002-9394(01)01218-1

[32] Rueden CT, Schindelin J, Hiner MC, et al. ImageJ2: ImageJ for the next generation of scientific image data. *BMC Bioinformatics*. 2017; 18: 529. 29187165 10.1186/s12859-017-1934-z PMC5708080

[33] Liefers B, Colijn JM, González-Gonzalo C, et al. A deep learning model for segmentation of geographic atrophy to study its long-term natural history. *Ophthalmology*. 2020; 127: 1086–1096. 32197912 10.1016/j.ophtha.2020.02.009

[34] McGraw KO, Wong SP. Forming inferences about some intraclass correlation coefficients. *Psychol Methods*. 1996; 1: 30.

[35] Bernardes R, Serranho P, Lobo C. Digital ocular fundus imaging: a review. *Ophthalmologica*. 2011; 226: 161–181. 21952522 10.1159/000329597

[36] Del Priore LV, Kuo Y-H, Tezel TH. Age-related changes in human RPE cell density and apoptosis proportion in situ. *Invest Ophthalmol Vis Sci*. 2002; 43: 3312–3318. 12356840

[37] Curcio CA, Allen KA, Sloan KR, et al. Distribution and morphology of human cone photoreceptors stained with anti-blue opsin. *J Comp Neurol*. 1991; 312: 610–624. 1722224 10.1002/cne.903120411

[38] Hoseini-Yazdi H, Vincent SJ, Collins MJ, Read SA, Alonso-Caneiro D. Wide-field choroidal thickness in myopes and emmetropes. *Sci Rep*. 2019; 9: 3474. 30837507 10.1038/s41598-019-39653-w PMC6401121

[39] Curcio CA, Sloan KR, Kalina RE, Hendrickson AE. Human photoreceptor topography. *J Comp Neurol*. 1990; 292: 497–523. 2324310 10.1002/cne.902920402

[40] Sunness JS, Bressler NM, Tian Y, Alexander J, Applegate CA. Measuring geographic atrophy in advanced age-related macular degeneration. *Invest Ophthalmol Vis Sci*. 1999; 40: 1761–1769. 10393046

[41] Nittala MG, Uji A, Velaga SB, et al. Effect of human central nervous system stem cell subretinal transplantation on progression of geographic atrophy secondary to nonneovascular age-related macular degeneration. *Ophthalmol Retina*. 2021; 5: 32–40. 32562884 10.1016/j.oret.2020.06.012

[42] Mauschitz MM, Fonseca S, Chang P, et al. Topography of geographic atrophy in age-related macular degeneration. *Invest Ophthalmol Vis Sci*. 2012; 53: 4932–4939. 22661483 10.1167/iovs.12-9711

[43] Sayegh RG, Sacu S, Dunavolgyi R, et al. Geographic atrophy and foveal-sparing changes related to visual acuity in patients with dry age-related macular degeneration over time. *Am J Ophthalmol*. 2017; 179: 118–128. 28385474 10.1016/j.ajo.2017.03.031

[44] Moult EM, Alibhai AY, Lee B, et al. A framework for multiscale quantitation of relationships between choriocapillaris flow impairment and geographic atrophy growth. *Am J Ophthalmol*. 2020; 214: 172–187. 31843474 10.1016/j.ajo.2019.12.006 PMC7951042

[45] Schmidt-Erfurth U, Bogunovic H, Grechenig C, et al. Role of deep learning-quantified hyperreflective foci for the prediction of geographic atrophy progression. *Am J Ophthalmol*. 2020; 216: 257–270. 32277942 10.1016/j.ajo.2020.03.042

[46] Jackson GR, Owsley C, Curcio CA. Photoreceptor degeneration and dysfunction in aging and age-related maculopathy. *Ageing Res Rev*. 2002; 1: 381–396. 12067593 10.1016/s1568-1637(02)00007-7

[47] Curcio CA, Owsley C, Jackson GR. Spare the rods, save the cones in aging and age-related maculopathy. *Invest Ophthalmol Vis Sci*. 2000; 41: 2015–2018. 10892836

[48] Curcio CA, Medeiros NE, Millican CL. Photoreceptor loss in age-related macular degeneration. *Invest Ophthalmol Vis Sci*. 1996; 37: 1236–1249. 8641827

[49] Curcio CA. Photoreceptor topography in ageing and age-related maculopathy. *Eye (Lond)*. 2001; 15: 376–383. 11450761 10.1038/eye.2001.140

[50] Owsley C, Jackson GR, Cideciyan AV, et al. Psychophysical evidence for rod vulnerability in age-related macular degeneration. *Invest Ophthalmol Vis Sci*. 2000; 41: 267–273. 10634630

[51] Pfau M, Müller PL, von der Emde L, et al. Mesopic and dark-adapted two-color fundus-controlled perimetry in geographic atrophy secondary to age-related macular degeneration. *Retina*. 2020; 40: 169–180. 30300264 10.1097/IAE.0000000000002337

[52] Beatty S, Murray IJ, Henson DB, Carden D, Koh H-H, Boulton ME. Macular pigment and risk for age-related macular degeneration in subjects from a Northern European population. *Invest Ophthalmol Vis Sci*. 2001; 42: 439–446. 11157880

[53] Pollreisz A, Reiter GS, Bogunovic H, et al. Topographic distribution and progression of soft drusen volume in age-related macular degeneration implicate neurobiology of fovea. *Invest Ophthalmol Vis Sci*. 2021; 62: 26. 10.1167/iovs.62.2.26 PMC7900846 33605982

[54] Kar D, Clark ME, Swain TA, et al. Local abundance of macular xanthophyll pigment is associated with rod- and cone-mediated vision in aging and age-related macular degeneration. *Invest Ophthalmol Vis Sci*. 2020; 61: 46. 10.1167/iovs.61.8.46 PMC7425747 32729911

[55] Nassisi M, Baghdasaryan E, Tepelus T, Asanad S, Borrelli E, Sadda SR. Topographic distribution of choriocapillaris flow deficits in healthy eyes. *PLoS One*. 2018; 13: e0207638. 30440050 10.1371/journal.pone.0207638 PMC6237387

[56] Gendelman I, Alibhai AY, Moult EM, et al. Topographic analysis of macular choriocapillaris flow deficits in diabetic retinopathy using swept-source optical coherence tomography angiography. *Int J Retina Vitreous*. 2020; 6: 6. 32206342 10.1186/s40942-020-00209-0 PMC7081691

[57] Zheng F, Zhang Q, Shi Y, et al. Age-dependent changes in the macular choriocapillaris of normal eyes imaged with swept-source optical coherence tomography angiography. *Am J Ophthalmol*. 2019; 200: 110–122. 30639367 10.1016/j.ajo.2018.12.025 PMC6513331

[58] Curcio CA, Allen KA. Topography of ganglion cells in human retina. *J Comp Neurol*. 1990; 300: 5–25. 2229487 10.1002/cne.903000103

[59] Liu T, Jung H, Liu J, Droettboom M, Tam J. Noninvasive near infrared autofluorescence imaging of retinal pigment epithelial cells in the human retina using adaptive optics. *Biomed Opt Express*. 2017; 8: 4348–4360. 29082069 10.1364/BOE.8.004348 PMC5654784

[60] Bermond K, Wobbe C, Tarau IS, et al. Autofluorescent granules of the human retinal pigment epithelium: phenotypes, intracellular distribution, and age-related topography. *Invest Ophthalmol Vis Sci*. 2020; 61: 35. 10.1167/iovs.61.5.35 PMC7405767 32433758

[61] Anderson DMG, Messinger JD, Patterson NH, et al. Lipid landscape of the human retina and supporting tissues revealed by high-resolution imaging mass spectrometry. *J Am Soc Mass Spectrom*. 2020; 31: 2426–2436. 32628476 10.1021/jasms.0c00119 PMC8161663

[62] Cooper RF, Wilk MA, Tarima S, Carroll J. Evaluating descriptive metrics of the human cone mosaic descriptive metrics of the human cone mosaic. *Invest Ophthalmol Vis Sci*. 2016; 57: 2992–3001. 27273598 10.1167/iovs.16-19072 PMC4898203

[63] Chen L, Li M, Messinger JD, Ferrara D, Curcio CA, Freund KB. Recognizing atrophy and mixed-type neovascularization in age-related macular degeneration via clinicopathologic correlation. *Transl Vis Sci Technol*. 2020; 9: 8. 10.1167/tvst.9.8.8 PMC7422865 32855855

[64] Guymer RH, Rosenfeld PJ, Curcio CA, et al. Incomplete retinal pigment epithelial and outer retinal atrophy in age-related macular degeneration: Classification of Atrophy Meeting Report 4. *Ophthalmology*. 2020; 127: 394–409. 31708275 10.1016/j.ophtha.2019.09.035 PMC7218279

[65] Chen L, Messinger JD, Ferrara D, Freund KB, Curcio CA. Stages of drusen-associated atrophy in age-related macular degeneration visible via histologically validated fundus autofluorescence. *Ophthalmol Retina*. 2020. 10.1016/j.oret.2020.11.006 PMC9749404 33217617

[66] Fritsche LG, Igl W, Bailey JNC, et al. A large genome-wide association study of age-related macular degeneration highlights contributions of rare and common variants. *Nat Genet*. 2016; 48: 134–143. 26691988 10.1038/ng.3448 PMC4745342

[67] Seddon JM, Widjajahakim R, Rosner B. Rare and common genetic variants, smoking, and body mass index: progression and earlier age of developing advanced age-related macular degeneration. *Invest Ophthalmol Vis Sci*. 2020; 61: 32–32. 10.1167/iovs.61.14.32 PMC7774056 33369641

[68] Seddon JM, Francis PJ, George S, Schultz DW, Rosner B, Klein ML. Association of CFH Y402H and LOC387715 A69S with progression of age-related macular degeneration. *J Am Med Assoc*. 2007; 297: 1793–1800. 10.1001/jama.297.16.1793 17456821

[69] Klein ML, Ferris FL, Francis PJ, et al. Progression of geographic atrophy and genotype in age-related macular degeneration. *Ophthalmology*. 2010; 117: 1554–1559.e1551. 20381870 10.1016/j.ophtha.2009.12.012 PMC2904435

[70] Grassmann F, Fleckenstein M, Chew EY, et al. Clinical and genetic factors associated with progression of geographic atrophy lesions in age-related macular degeneration. *PLoS One*. 2015; 10: e0126636. 25962167 10.1371/journal.pone.0126636 PMC4427438

[71] Wong WT, Kam W, Cunningham D, et al. Treatment of geographic atrophy by the topical administration of OT-551: results of a phase II clinical trial. *Invest Ophthalmol Vis Sci*. 2010; 51: 6131–6139. 20574018 10.1167/iovs.10-5637 PMC3055748

[72] Domalpally A, Danis R, Agron E, Blodi B, Clemons T, Chew E. Evaluation of geographic atrophy from color photographs and fundus autofluorescence images: Age-Related Eye Disease Study 2 report number 11. *Ophthalmology*. 2016; 123: 2401–2407. 27448832 10.1016/j.ophtha.2016.06.025 PMC5077673

[73] Yaspan BL, Williams DF, Holz FG, et al. Targeting factor D of the alternative complement pathway reduces geographic atrophy progression secondary to age-related macular degeneration. *Sci Transl Med*. 2017; 9: eaaf1443. 28637922 10.1126/scitranslmed.aaf1443

[74] Holz FG, Bindewald-Wittich A, Fleckenstein M, Dreyhaupt J, Scholl HPN, Schmitz-Valckenberg S. Progression of geographic atrophy and impact of fundus autofluorescence patterns in age-related macular degeneration. *Am J Ophthalmol*. 2007; 143: 463–472.e462. 17239336 10.1016/j.ajo.2006.11.041

[75] Shen LL, Liu F, Nardini HG, Del Priore LV. Reclassification of fundus autofluorescence patterns surrounding geographic atrophy based on progression rate: a systematic review and meta-analysis. *Retina*. 2019; 39: 1829–1839. 30829988 10.1097/IAE.0000000000002480

[76] Park MM, Young BK, Shen LL, Adelman RA, Del Priore LV. Topographic variation of retinal vascular density in normal eyes using optical coherence tomography angiography. *Trans Vis Sci Tech.* 2021; 10(12): 15. 10.1167/tvst.10.12.15 PMC8525867 34647965

