# Supplementary material for: Local Progression Kinetics of Geographic Atrophy Depends Upon the Border Location
Source: Invest Ophthalmol Vis Sci. 2021 Oct 28;62(13):28. doi: 10.1167/iovs.62.13.28 (PMC8558522; doi:10.1167/iovs.62.13.28)
Supplement: Supplement 1 [file iovs-62-13-28_s001.pdf]

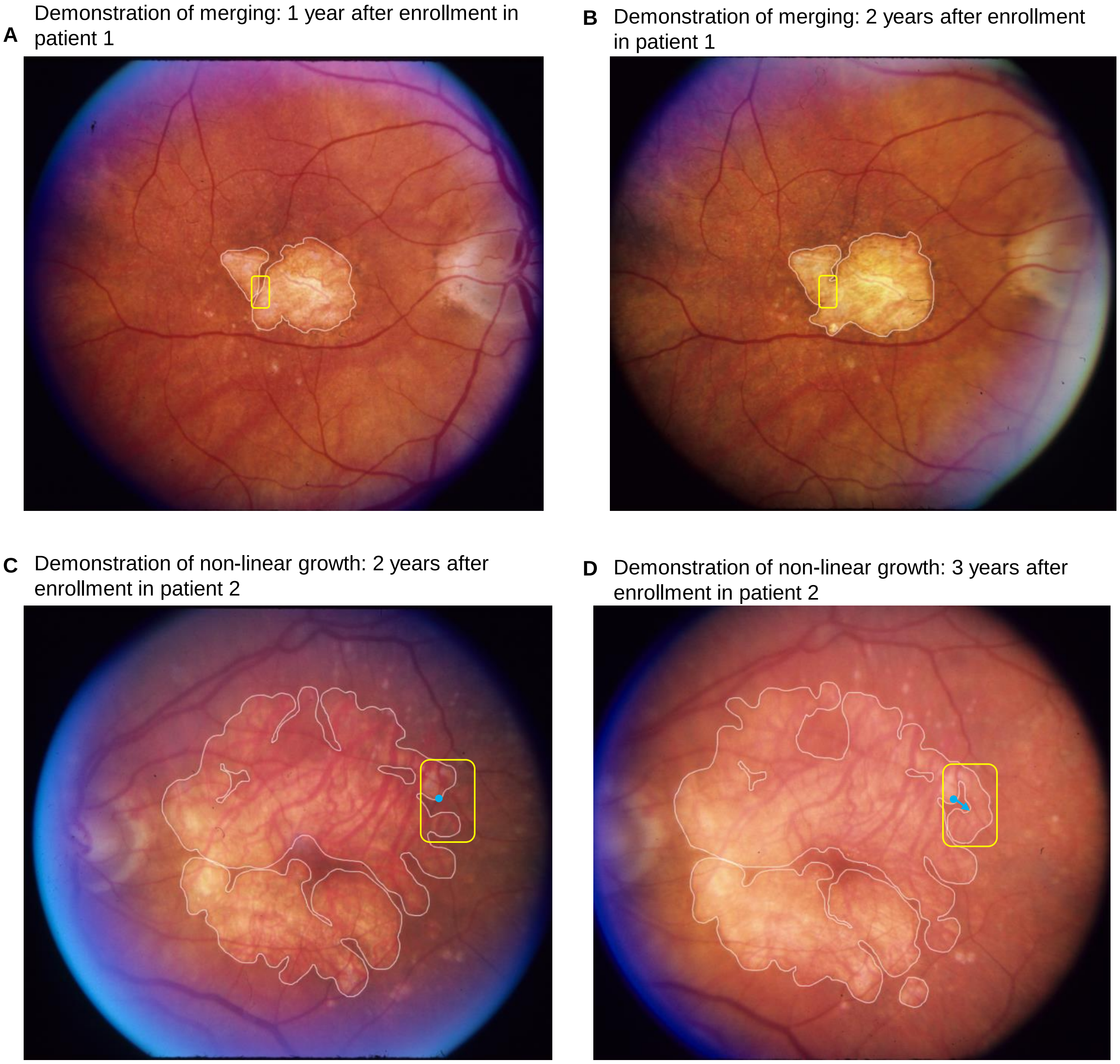

Supplementary Figure S1. Scenarios where geographic atrophy (GA) border expansion rate (BER) cannot be measured reliably. **A** and **B**, Demonstration of GA merging. Patient 1 had 2 GA lesions 1 year after enrollment into the study (**A**). Over 1 year, the 2 GA lesions merged into 1 (**B**). We could not calculate GA BER in the marked region (yellow rectangle in **B**) since GA margin in this region disappeared at the follow-up visit. Among 237 eyes included in the study, 66 eyes had GA merging over 1 year. **C** and **D**, Demonstration of non-linear growth of GA lesion. In the region marked by the yellow rectangle, GA border did not expand linearly. The non-linear GA growth could be because GA margin progressed along a curved path, or a new GA lesion developed in this region. Since GA BER was defined as the shortest distance from a pixel in visit 2 to GA border in visit 1, the measurement of GA BER in some pixels (e.g., marked by the blue dot and blue arrow) would pass through a non-atrophic region, which could not reflect the actual GA progression rate. Among 237 eyes included in the study, only 8 eyes had non-linear GA growth.
