# Supplementary material for: Local Progression Kinetics of Geographic Atrophy Depends Upon the Border Location
Source: Invest Ophthalmol Vis Sci. 2021 Oct 28;62(13):28. doi: 10.1167/iovs.62.13.28 (PMC8558522; doi:10.1167/iovs.62.13.28)
Supplement: Supplement 2 [file iovs-62-13-28_s002.pdf]

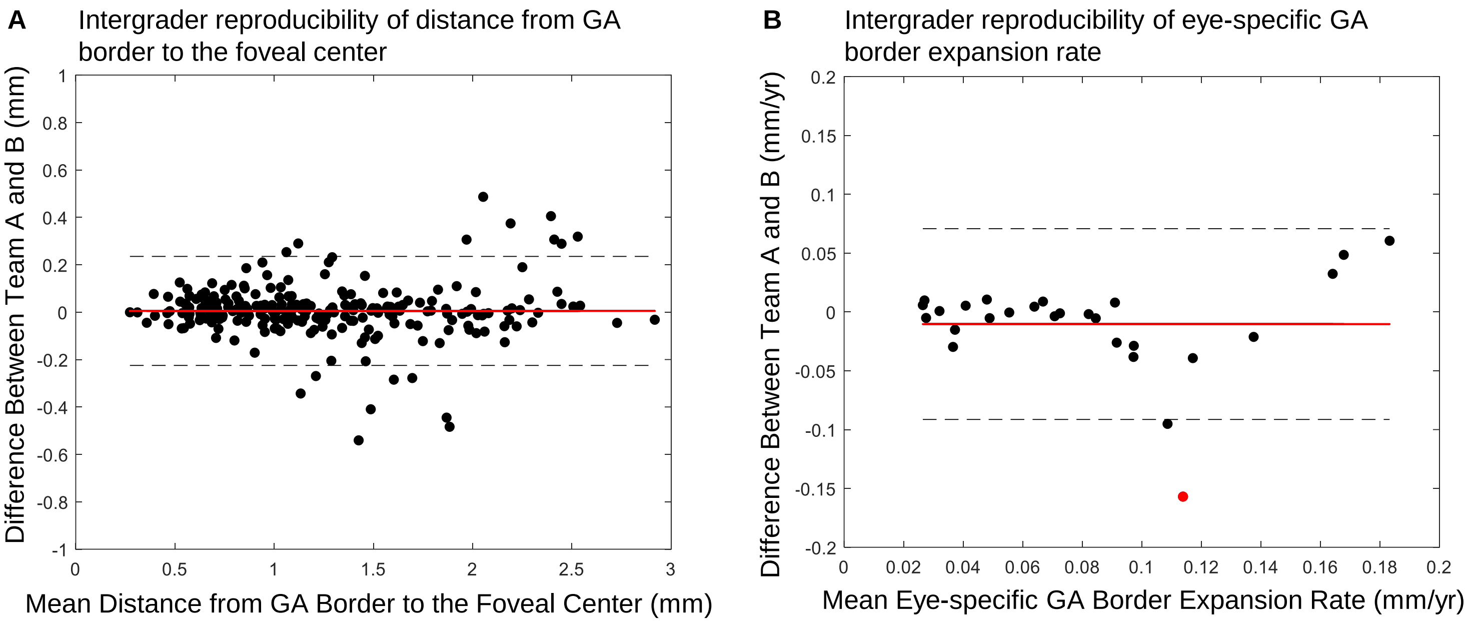

Supplementary Figure S2. Intergrader reproducibility of geographic atrophy (GA) grading. **A**, Intergrader reproducibility of the mean distance from GA border to the foveal center (N = 240 visits). Team A and B had a mean difference of 0.01 mm (the solid red line) and 95% limits of agreement of -0.23 to +0.24 mm (dashed black lines). The intraclass correlation coefficient (ICC) was 0.98. **B**, Intergrader reproducibility of eye-specific GA border expansion rate (N = 27 eyes). We calculated the eye-specific GA border expansion rate as the mean local border expansion rate of all points on GA border in each eye over 1 year. Team A and B had a mean difference of -0.01 mm/year (the solid red line) and 95% limits of agreement of -0.091 to +0.071 mm/year (dashed black lines). The ICC was 0.64 and it increased to 0.81 after we removed 1 outlier from the analysis (red circle; shown in Supplementary Figure S3).
