# Supplementary material for: Local Progression Kinetics of Geographic Atrophy Depends Upon the Border Location
Source: Invest Ophthalmol Vis Sci. 2021 Oct 28;62(13):28. doi: 10.1167/iovs.62.13.28 (PMC8558522; doi:10.1167/iovs.62.13.28)
Supplement: Supplement 3 [file iovs-62-13-28_s003.pdf]

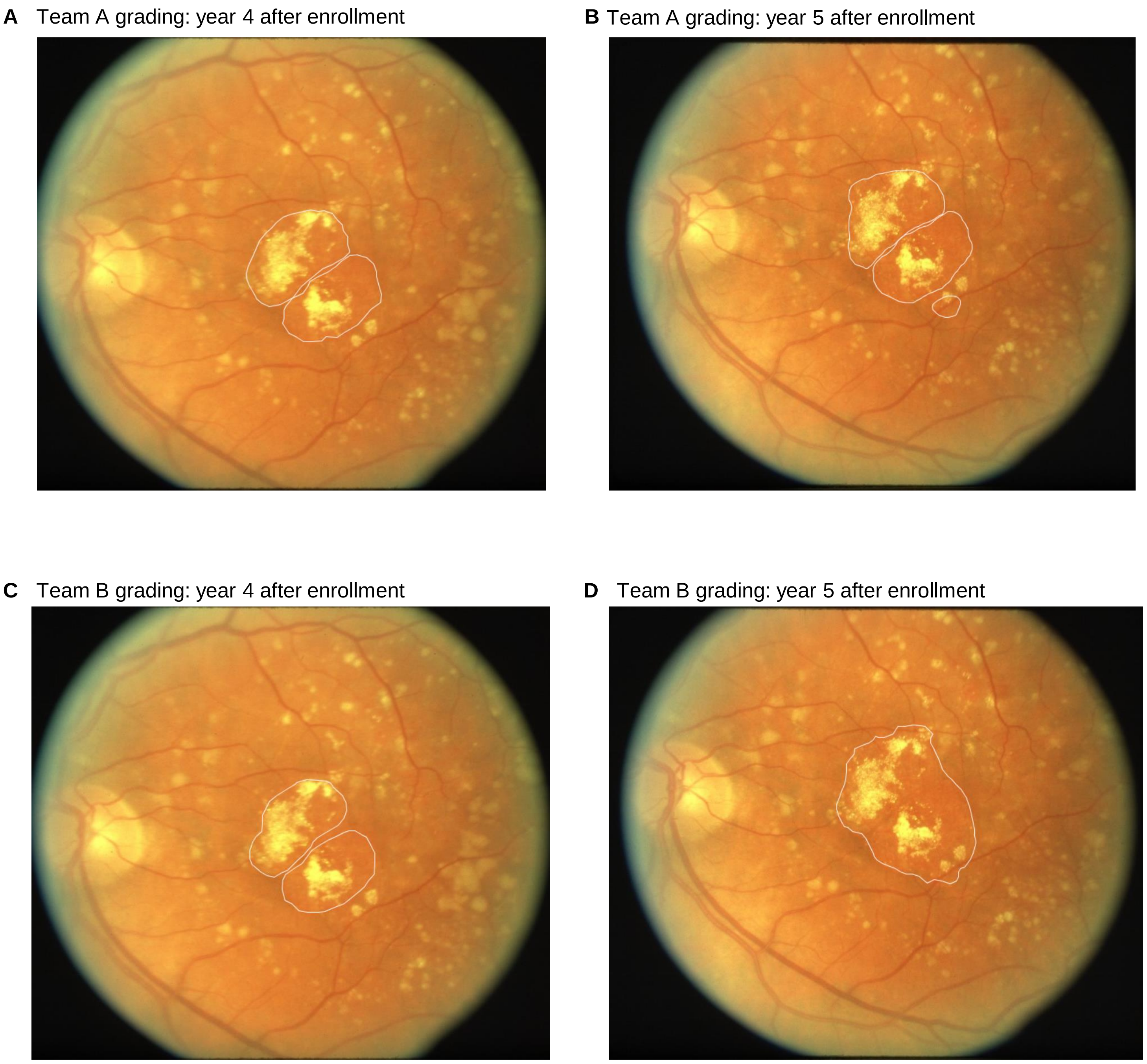

Supplementary Figure S3. Demonstration of an eye in which eye-specific geographic atrophy (GA) border expansion rate (BER) differed between team A (**A** and **B**) and team B (**C** and **D**). **A** and **C**, GA delineations at year 4 after enrollment were comparable between team A and B. However, team A and B differed greatly at year 5 after enrollment, which was likely because GA margins were unclear in the color fundus photograph. Due to the measurement errors, eye-specific GA BER was 0.04 mm/year based on team A's gradings but was 0.19 mm/year based on team B's gradings.
