# Supplementary material for: Local Progression Kinetics of Geographic Atrophy Depends Upon the Border Location
Source: Invest Ophthalmol Vis Sci. 2021 Oct 28;62(13):28. doi: 10.1167/iovs.62.13.28 (PMC8558522; doi:10.1167/iovs.62.13.28)
Supplement: Supplement 4 [file iovs-62-13-28_s004.pdf]

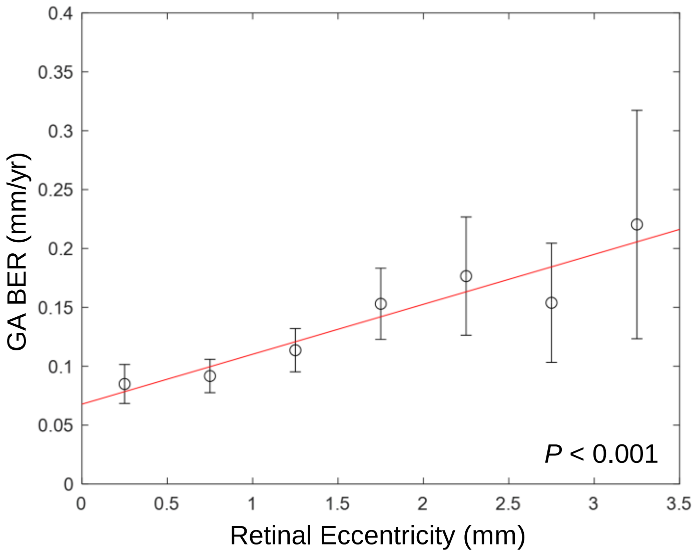

Supplementary Figure S4. Local geographic atrophy (GA) border expansion rate (BER) increased as a function of retinal eccentricity after we removed GA lesions that had any GA margins merging (66 eyes) or non-linear growth (8 eyes) ($P < 0.001$). The total number of eyes in the analysis was 169. GA BER was 0.09 mm/year (95% CI = 0.068 to 0.10; N = 99 eyes) in zone 1 and increased by 2.6-fold to 0.22 mm/year (95% CI = 0.12 to 0.32; N = 26 eyes) in zone 7.
