# Supplementary material for: Local Progression Kinetics of Geographic Atrophy Depends Upon the Border Location
Source: Invest Ophthalmol Vis Sci. 2021 Oct 28;62(13):28. doi: 10.1167/iovs.62.13.28 (PMC8558522; doi:10.1167/iovs.62.13.28)
Supplement: Supplement 5 [file iovs-62-13-28_s005.pdf]

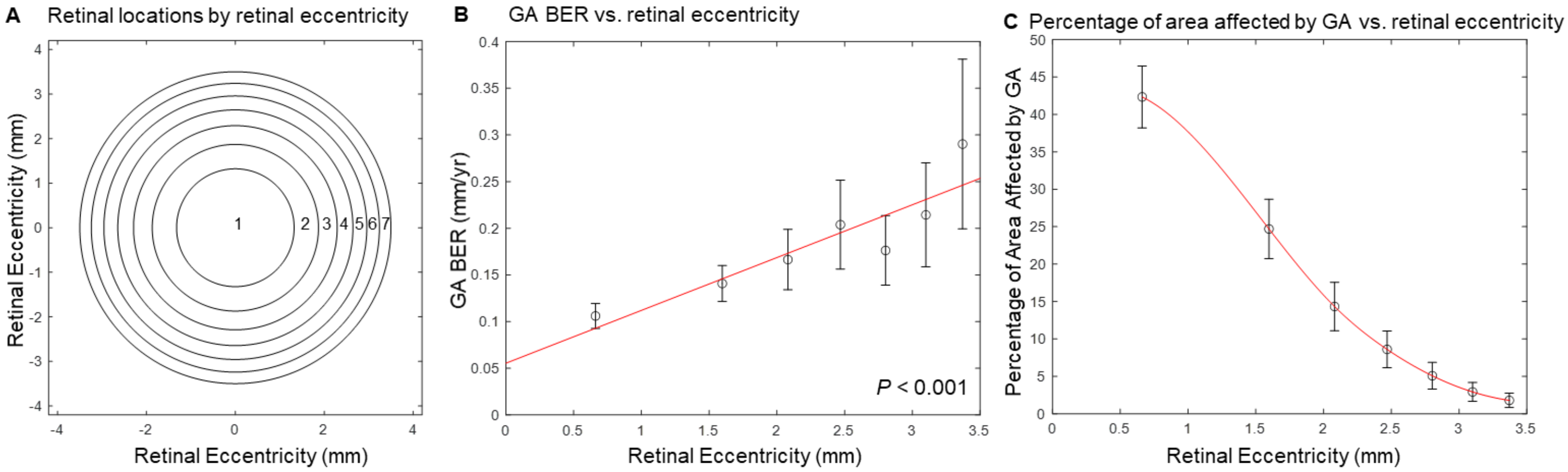

Supplementary Figure S5. Variation of geographic atrophy (GA) progression and distribution in zones with same areas at different retinal eccentricities (N = 237 eyes). The error bar represents 95% confidence interval (CI) for the mean. The red line represents the trendline. **A**, We divided the retina into 7 topographic zones with the same areas (5.5 mm^2) using concentric circles (centered at the fovea) from 0 to 3.5 mm retinal eccentricity. **B**, Local GA border expansion rate (BER) increased progressively as a function of retinal eccentricity ($P < 0.001$). **C**, The percentage of retinal area affected by GA was calculated as GA area in each zone divided by total retinal area in the corresponding zone. As the retinal eccentricity increased from 0 to 3.5 mm, the percentage of retinal area affected by GA decreased progressively. These results were consistent with the results shown in Figure 3.
