# Supplementary material for: Local Progression Kinetics of Geographic Atrophy Depends Upon the Border Location
Source: Invest Ophthalmol Vis Sci. 2021 Oct 28;62(13):28. doi: 10.1167/iovs.62.13.28 (PMC8558522; doi:10.1167/iovs.62.13.28)
Supplement: Supplement 6 [file iovs-62-13-28_s006.pdf]

A Demonstration of GA expansion

B GA enlargement area over an infinitely short time period

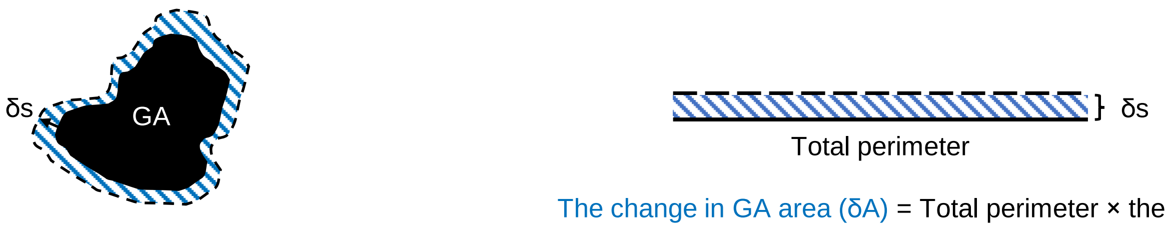

The change in GA area (δA) = Total perimeter × the linear expansion of GA border (δs)

GA perimeter-adjusted growth rate = $\frac{\delta A}{Total\ Perimeter}$

GA border expansion rate = δs

Therefore, GA perimeter-adjusted growth rate = GA border expansion rate over an infinitely short time period

Supplementary Figure S6. Demonstration of the anatomic meaning of perimeter-adjusted growth rate of geographic atrophy (GA) and eye-specific GA border expansion rate (BER). **A**, The change in GA area (δA) over an infinitely short time period is a thin ring (shaded blue) with an infinitely small linear growth (δs). **B**, Based on the principle of calculus, an infinitesimally thin ring is the same as a rectangle and the change in GA area (δA) can be calculated as the Total Perimeter × δs. GA perimeter-adjusted growth rate is defined as GA area growth rate/mean total GA perimeter between the first and last visit, which is $\frac{\delta A}{Total\ Perimeter}$ in this case. The eye-specific GA BER is the direct measurement of the mean linear expansion rate of GA border, which is δs in this example. Since $\frac{\delta A}{Total\ Perimeter}$ = δs over an infinitely short time period, GA perimeter-adjusted growth rate equals to the eye-specific BER under this ideal circumstance (i.e., precisely delineated GA border and infinitely small follow-up duration). But in reality, the 2 parameters are influenced by different measurement errors and the follow-up duration. GA perimeter-adjusted growth rate is influenced by measurement errors in GA area and perimeter, whereas eye-specific GA BER is affected by measurement errors in the linear distance determined from the Euclidean distance map.
