# Supplementary material for: Local Progression Kinetics of Geographic Atrophy Depends Upon the Border Location
Source: Invest Ophthalmol Vis Sci. 2021 Oct 28;62(13):28. doi: 10.1167/iovs.62.13.28 (PMC8558522; doi:10.1167/iovs.62.13.28)
Supplement: Supplement 7 [file iovs-62-13-28_s007.pdf]

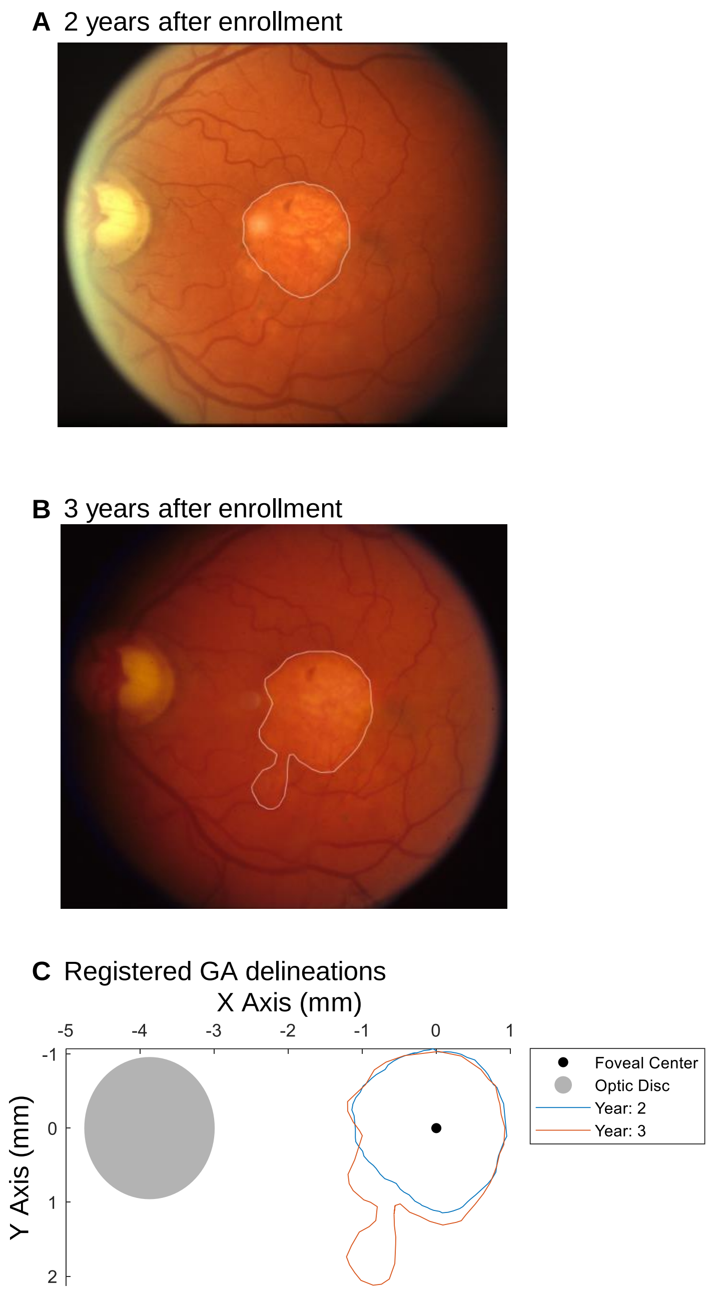


**Supplementary Figure S7.** Demonstration of a case where eye-specific GA border expansion rate (BER) differs from GA perimeter-adjusted growth rate. **A**, GA delineation at 2 years after enrollment. **B**, GA delineation after an additional year of follow-up. **C**, Registered GA delineations of the 2 visits. In this eye, eye specific GA BER was 0.29 mm/year but GA perimeter-adjusted growth rate was only 0.12 mm/year. The difference occurred when GA delineation at the follow-up visit had a small GA border protrusion. The border protrusion caused a higher linear distance measurement of GA expansion than the increase in GA area measurement. The small GA border protrusion could occur due to actual GA growth at the border, the onset of new GA lesion near the GA margin, or measurement error.
