# Supplementary material for: Local Progression Kinetics of Geographic Atrophy Depends Upon the Border Location
Source: Invest Ophthalmol Vis Sci. 2021 Oct 28;62(13):28. doi: 10.1167/iovs.62.13.28 (PMC8558522; doi:10.1167/iovs.62.13.28)
Supplement: Supplement 8 [file iovs-62-13-28_s008.pdf]

Supplementary Table S1. Baseline Characteristics of the Study Cohort

Patients (eyes)	160 (237)
Age of patients, years, mean (SD)	70.5 (5.3)
Sex, male, n (%)	88 (55.0)
Baseline GA area, mm^2, mean (SD)	5.8 (7.7)
Baseline GA perimeter, mm, mean (SD)	11.7 (10.7)
Baseline GA circularity index, mean (SD)	0.61 (0.30)
Baseline unifocal GA, number of eyes (%)	153 (64.6)
Baseline number of lesions, mean (SD)	1.9 (1.7)
Baseline distance between GA border and the foveal center, mm, mean (SD)	1.3 (0.6)
Baseline GA in the fellow eye, number of eyes (%)	156 (65.8)

GA, geographic atrophy; SD, standard deviation
